# Quantitative parameters of productive transcription on T5 N25–based promoters are modulated by the initial transcribed sequence and template supercoiling

**DOI:** 10.1016/j.jbc.2025.110610

**Published:** 2025-08-25

**Authors:** Lilian M. Hsu, N. Natalie Han

**Affiliations:** Program in Biochemistry, Mount Holyoke College, South Hadley, Massachusetts, USA

**Keywords:** bacterial transcription, promoter, enzyme kinetics, enzyme mechanism, abortive initiation, promoter escape, scrunching, initial transcribed sequence, supercoiling

## Abstract

Productive initiation on the escape rate–limited T5 phage *N25* promoter is subject to substantial modulation by the initial transcribed sequence (ITS). It is further compromised by the formation of two classes of open complexes—productive and unproductive. To decipher their roles, we performed single-cycle transcription assays under RNA polymerase (RNAP)–limiting conditions to quantitatively determine the rate of promoter escape and the productive fraction of RNAP open complexes formed at four *N25-ITS* variant promoters. Each promoter variant was transcribed from three different template conformations, that is, two fragment templates of different lengths and a supercoiled plasmid DNA template. In addition, each time-course transcription reaction was performed in parallel without or with GreB. Our results indicate that ITS variation greatly impacts both parameters, which together determine the extent of productive RNA synthesis from a promoter. Further, both parameters are highly stimulated by template supercoiling, which yields a higher fraction of productive complexes that undergo promoter escape at a faster rate. In contrast, the effect of GreB is selective, showing little effect on RNAP partitioning but increasing the escape rate of *N25* variants bearing non-native ITSs. Analysis of the abortive RNA synthesis kinetics on the highly abortive *N25anti(-A)* promoter reveals the existence of an unproductive ITC making a 7-nt abortive RNA continuously. Based on our new kinetic data and recently published structural information on promoter complexes, we propose for T5 *N25* promoters a mechanism of transcription initiation-promoter escape consistent with the roles of the ITS and template supercoiling.

Transcriptional gene expression is controlled and specified by DNA signals known as promoters. To initiate transcription, an RNA polymerase (RNAP) molecule must first recognize and bind the double-stranded promoter DNA to form a closed complex (RPc), then melt open a stretch of DNA to form a transcription bubble within an open complex (RPo), which is now catalytically active and competent at transcription initiation (1). The two-step process of RPo formation can be described by an equilibrium binding constant *K*_*B*_ (=*k*_*1*_/*k*_−*1*_) for the first step and a forward (melting) isomerization rate constant, *k*_*2*_, for the second step. These constants have been obtained using a steady-state abortive initiation assay (2). The product *K*_*B*_^.^*k*_*2*_ is directly correlated with the initiation frequency at a promoter; a high *K*_*B*_^.^*k*_*2*_ value represents high *in vitro* promoter strength. Among 31 promoters examined, the *K*_*B*_^.^*k*_*2*_ value can range over four orders of magnitude (3).

For *Escherichia coli* RNAP holoenzyme (Eσ^70^), a minimal promoter DNA fragment of ∼70 bps (recovered from DNase I protection footprint of an RPo) spans the nucleotide sequence from −50 to +20, relative to +1, the start site of transcription (4). By aligning many promoter sequences at their +1 position, a core promoter structure—composed of two highly conserved hexameric elements around the −35 and −10 positions—emerged over time to show a consensus sequence of −35 box [TTGACA]/17-bp spacer/−10 box [TATAAT] (5, 6, 7, 8). A consensus promoter displays the highest *K*_*B*_^.^*k*_*2*_ value (because of the direct involvement of the −35 and −10 box sequences in binding interaction with RNAP during RPo formation); yet no natural Eσ^70^ promoter adopts this sequence. This observation led to the conclusion that transcriptional gene expression in *E. coli* is optimized, rather than maximized, for biological function (9). To optimize a core promoter that deviates from the consensus, several other polymerase-binding elements—including the upstream activating element (UP) around −43, the extended −10 element TGn at −15 to −13, and the G_−7_/G_−5_ residue in the 8-/6-bp discriminator (DIS)—have been found to be involved, in a mix-and-match fashion, to bolster each individual promoter’s function ((10), and references cited within).

When investigating the activity of a promoter, we are interested in understanding two issues. One is how does a promoter achieve its strength? Two is how is a promoter regulated? The primary determinant of promoter strength is the promoter sequence itself and how closely it agrees with the consensus sequence. The product *K*_*B*_^.^*k*_*2*_ has been shown to be a measure of promoter strength *in vitro*, but is it also a measure of promoter strength *in vivo* (judging by the level of gene product formed)? Brunner and Bujard (11) investigated this question by examining a group of phage “early” promoters—T5 *N25*, T7 *A1*, and λ *P*_*L*_, among others—and determined their *k*_on_ (similar to *K*_*B*_^.^*k*_*2*_ but determined by a filter binding assay), *t*_1/2_ (open complex half-life, which is the inverse of *k*_off_), *K*_eq_ (= *k*_on_/*k*_off_), and as well, the relative level of the reporter β-lactamase activity (indexed to the level primed by *P*_bla_). Their results ranked T5 *N25* > T7 *A1* > λ *P*_*L*_ for *in vitro* promoter strength but T7 *A1* > λ *P*_*L*_ > T5 *N25* for *in vivo* productive transcription. The discrepancy between *in vitro* and *in vivo* measures of promoter strength brought into focus the latter stage of transcription initiation—which entails the steps between RNA chain initiation and the enzyme’s transition into the elongation phase—and prompted further studies of the T5 *N25* promoter to uncover the cause of the discrepancy.

T5 *N25* is an *E. coli* Eσ^70^ promoter that binds RNAP to form a highly stable open complex (*k*_on_ = 2.9 × 10^8^ M^−1^ s^−1^) with an extremely long half-life (*t*_1/2_ = 180 min), yielding a *K*_eq_ = 4.5 × 10^12^ M^−1^ (11). Transcription initiation from such a promoter is rate limited at the escape step (11, 12). The manifestation of this rate limitation results in the repetitive synthesis of a set of abortive RNAs, ranging in size from 2 to 10 nt, whose sequences are specified by the initial transcribed sequence (ITS) (13). Bujard’s group further investigated the effect of changing the ITS (from +3 to +20) to a version called *antiDSR* and found that *N25*_*antiDSR*_ now produces full-length (FL) RNA at 1/10th the level of *N25* (14). The greatly reduced level of productive RNA synthesis came with a highly elevated level of abortive initiation, yielding an expanded collection of abortive RNAs ranging from 2 to 14 nt (13). Broadening this line of investigation to 43 ITSs, we found that, in general, changing the ITS greatly altered the abortive-productive transcription properties of the *N25* promoters (15). Depending on the ITS, *N25*-ITS variants undergo different abortive transcription programs, and their productive efficiency can vary by as much as 25-fold (15). Simply put, the ITS can alter the promoter escape barrier for the individual *N25* promoters and affect FL RNA synthesis. But how an ITS exerts its influence during promoter escape remains unanswered.

The role of ITS in productive gene expression has been vastly extended in a comprehensive study of 384 promoter–ITS combinations generated by adapting each of 96 ITSs to four *E. coli* promoters—λ *P*_*R*_, *lac*UV5, *deo*B, and *acn*B (16). Using a massively parallel next-generation sequencing strategy for estimating the relative level of fast- and slow-escaping products, the authors found that the ITS can greatly modulate the productive output from each promoter—chiefly by affecting the rate of promoter escape, but the pattern of modulation is dependent on the promoter context. Thus, productive synthesis from the aforementioned promoters varied 50-, 144-, 106-, and 93-fold, respectively.

On the strong-binding promoters, there emerged a second factor—first shown with λ *P*_*R*_ and *lac*UV5 (17), then on T7 *A1* (18) and T5 *N25* (19)—that could influence productive RNA synthesis; that is, RNAP–promoter binding results in the formation of two classes of open complexes: productive and unproductive (19). The productive open complex can initiate transcription, synthesize a requisite series of abortive RNAs, and undergo the escape transition to the elongation phase to synthesize FL RNA. The unproductive open complex, on the other hand, can initiate transcription but cannot undergo escape and thus, is held back at the promoter to repeatedly synthesize the abortive RNAs (19). The biochemical demonstration of these two classes of open complex gave rise to the branched pathway model of transcription initiation (Fig. 1*A*). The branched pathway model was shown to be widely adopted by many *E. coli* promoters both *in vitro* and *in vivo* (18). *In vivo*, it has been shown on several candidate promoters to be a way of regulating gene expression *via* Gre factors (18). However, despite its wide occurrence, few studies have been conducted to examine the quantitative and qualitative aspects of the RNAP partitioning process.

**Figure 1:**
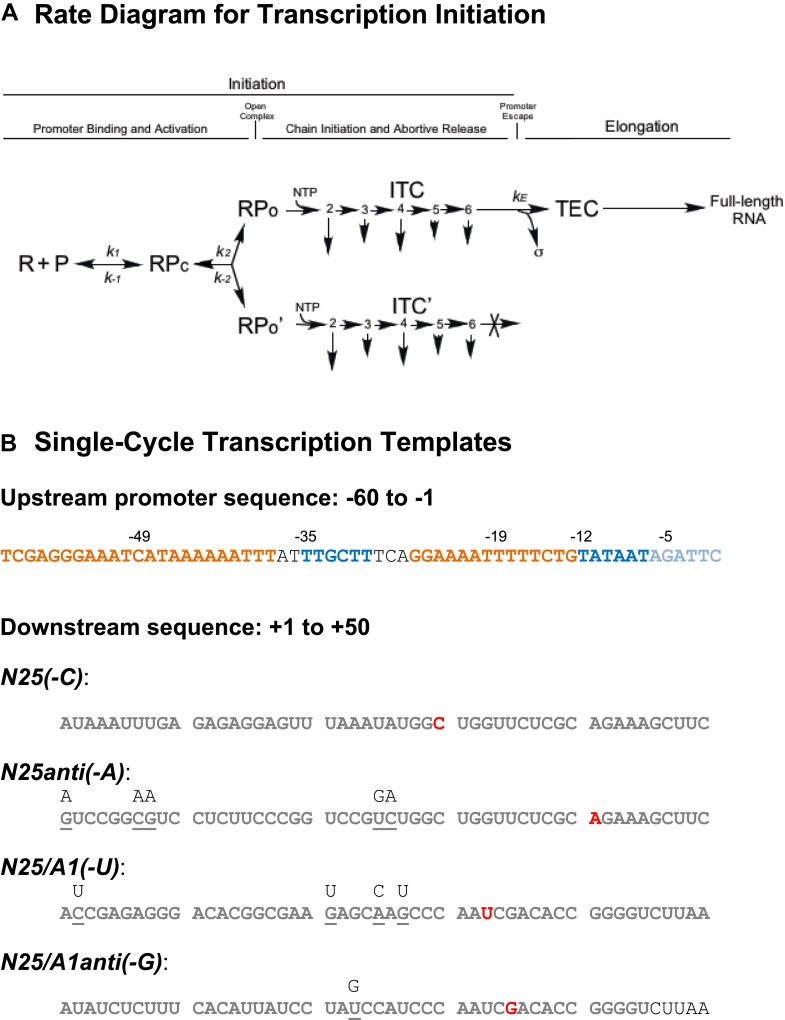
**Abranched pathway modelof transcriptioninitiation.***A*, rate diagram for transcription initiation. RNAP (R) binds to the promoter sequence (P) to form first a closed complex (RPc). The closed complex then “melts” (or isomerizes) to form two types of open complex (RPo and RPo’), both of which can perform *de novo* transcription in the presence of NTP, forming two types of initial transcribing complexes (ITC and ITC’), productive and unproductive, respectively. The ITC is competent at promoter escape after undergoing an obligate series of abortive initiation steps (indicated by *downward arrows*), whereas ITC’ is trapped to undergo abortive initiation continually. (For simplicity, only ITC and ITC’ bearing 2- to 6-nucleotide transcripts are represented in this diagram.) The constants *k*_*1*_ and *k*_−*1*_ govern the forward and reverse rates of closed complex formation and constants *k*_*2*_ and *k*_−*2*_, forward and reverse rates of isomerization. The constant *k*_*E*_ is a composite rate constant that encompasses all the reaction rates of nucleotide incorporation and abortive release during abortive initiation and promoter escape. In this diagram, we assume the concurrence of sigma factor (σ^70^) release upon the promoter escape transition. *B,* single-cycle transcription templates: only the nontemplate strand sequence is shown. All four starting promoters (*i.e*., *N25*, *N25anti*, *N25/A1*, and *N25/A1anti*) contain identical upstream promoter sequence from −60 to −1 (the −35 and −10 elements in *dark blue*, the canonical and spacer UP elements in *orange*, and the DIS in *light blue*). The promoters differ in their downstream sequence from +1 to +50; here, the *N25* and *N25anti* pair differs in sequence from +3 to +26, and the *N25/A1* and *N25/A1anti* pair differs in sequence from +3 to +20. A downstream sequence missing one of the four nucleotides was generated using a PCR primer. The replaced nucleotides are listed above the main sequence of *N25(-C)*, *N25anti(-A)*, *N25/A1(-U)*, and *N25/A1anti(-G).* In each case, the first occurrence of the missing nucleotide is highlighted in *bold red*. Thus, the full-length RNA expected at *N25(-C)* is C30; at *N25anti(-A*), A41; at *N25/A1(-U)*, U33; and at *N25/A1anti(-G)*, G35. RNAP, RNA polymerase; TEC, ternary elongation complex.

The goal of our study was to examine factors that affect the productive output from the T5 *N25*–based promoters. According to the branched pathway model of transcription initiation, there are two: one, RNAP partitioning into the productive *versus* unproductive open complexes (*i.e*., the productive fraction), and two, the rate of promoter escape (*i.e*., *k*_*E*_) from the productive complexes. Both parameters were quantitatively determined here from kinetic analysis of single-cycle transcription experiments performed under polymerase-limiting conditions. Below, we report the values obtained for four *N25–ITS* promoters, each in three template conformations, and the effect of including GreB in the reaction. We found that both parameters are significantly impacted by the ITS and by template supercoiling. Interpreting our kinetic data in light of the newly available structural details of many transcription complexes (20, 21, 22, 23) has fostered the creation of a new and plausible mechanism of transcription initiation–promoter escape for the T5 *N25* promoters.

## Results

### Promoters and template conformations

The four *N25–*ITS promoters we examined were *N25*, *N25anti*, *N25/A1*, and *N25/A1anti* (Fig. 1*B*). They were shown previously to undergo very different degrees of abortive-productive synthesis during steady-state transcription (15). *N25* contains the native ITS. *N25anti* was derived from *N25* by substituting A ↔ C and G ↔ T, the *anti*-algorithm, while avoiding triplet repeats at positions +3 to +20 of the *N25* ITS (24), which rendered *N25anti* ∼10-fold less effective than *N25* in productive RNA synthesis (13, 14). To determine if the potent inhibitory effect on promoter escape induced by the *anti*-ITS is a general phenomenon, we created the *N25/A1* and *N25/A1anti* pair (15). The *A1* ITS supports facile escape from its indigenous T7 *A1* promoter (13), a *P*_*RM*_ consensus promoter (25), as well as the T5 *N25* promoter (15). Changing the ITS of *N25/A1* using the *anti*-algorithm yielded *N25/A1anti* that also displayed severely impaired escape (15). The *anti*-algorithm has the effect of changing a highly purine-rich ITS on the nontemplate (NT) strand to a highly pyrimidine-rich one (15). The latter causes a bias for the pretranslocated state when the RNA 3′ dinucleotides are pyrimidines (26, 27, 28). In turn, the pretranslocated states of the pyrimidine-rich RNA are more prone to backtracking, resulting in abortive release of the nascent RNA and abrogating promoter escape (29, 30).

In anticipation of performing single-cycle transcription, which, for the *N25-*based promoters, could be reliably achieved by withholding one of four NTPs in the reaction and replacing the fourth nucleotide with its 3′ deoxy terminator analog, we made minimal modifications to the transcribed sequences of the aforementioned templates to obtain correspondingly *N25(-C)*, *N25anti(-A)*, *N25/A1(-U)*, and *N25/A1anti(-G)* (Fig. 1*B*). In each case, the base in parentheses corresponds to the terminator analog to be used with the template in transcriptional time-course reactions. Note that the transcribed sequence of *N25* is naturally devoid of C until position +30; thus, *N25(-C)* is identical to *N25*. Each promoter was then prepared in three or four configurations: as a short PCR-derived fragment (142 bp spanning −85 to +57; abbreviated PS), a longer PCR-derived fragment (348 bp spanning −234 to +114; abbreviated PL), a linearized plasmid DNA containing each promoter cloned into the pSA508 vector (31) (∼3560 bp linearized with XmnI to position the *N25* or related promoters centrally in the long fragment; abbreviated LN), or a supercoiled plasmid DNA (freshly isolated using Qiagen MidiPrep columns and treated with phenol–chloroform to remove residual RNase; abbreviated SC).

### Supercoiling facilitates promoter escape

By the steady-state transcription criterion, the modified promoter sequences all give rise to highly similar abortive-productive yields to those of the unmodified promoter DNAs analyzed in the short PS conformation (15). These results indicate that the minimal sequence alterations scattered in the initial transcribed region do not cause significant changes to the promoter escape properties. Having established this baseline, results of three independent trials of steady-state transcription and quantitation of abortive and productive yields, AY and PY, reveal that *N25(-C)* has a dramatic dependence on template conformation (Table 1). The productive yield is enhanced by increasing the length of the template DNA, reaching a very high level in the SC conformation. Relative to the PS template length, escape efficiency from the SC template is elevated ∼6-fold. *N25/A1(-U)*, on the other hand, appears to be largely unaffected by template conformation; escape is equally efficient in all conformations. For *N25anti(-A)* and *N25/A1anti(-G)*, only the SC conformation facilitates escape. Although the productive yields from these SC promoters are modest, they correspond to a 10- to 15-fold stimulation of escape relative to the PS conformation.

**Table 1:** Steady-state transcription parameters *versus* template conformation: Abortive and productive yields

Promoter	N25 (-C)		N25anti (-A)		N25/A1 (-U)		N25/A1anti (-G)	
Conformation	AY (%)	PY (%)	AY (%)	PY (%)	AY (%)	PY (%)	AY (%)	PY (%)
PS	94.4 ± 2.8	5.6 ± 2.8	99.7 ± 0.2	0.3 ± 0.2	90.4 ± 3.1	9.6 ± 3.1	99.6 ± 0.1	0.4 ± 0.1
PL	90.3 ± 1.4	9.7 ± 1.4	99.4 ± 0.1	0.6 ± 0.1	94.7 ± 3.7	5.3 ± 3.7	99.5 ± 0.1	0.5 ± 0.1
LN	83.2 ± 6.2	16.8 ± 6.2	99.2 ± 0.2	0.8 ± 0.2	94.7 ± 1.9	5.3 ± 1.9	99.6 ± 0.0	0.4 ± 0.0
SC	65.2 ± 22.6	34.8 ± 22.6	95.6 ± 1.1	4.4 ± 1.1	92.6 ± 3.6	7.4 ± 3.6	96.3 ± 0.8	3.7 ± 0.8

All numbers are averages of three to five independent experiments ± standard deviation.

### Kinetic analysis of escape

To measure the kinetics of escape, we performed the single-cycle transcription reaction in the presence of three NTPs and the 3′-deoxy analog of the fourth NTP to stall “FL” RNA synthesis at the position of the fourth base. Because the *N25* promoters all undergo many cycles of rapid abortive initiation before escape, we obtained a composite rate constant of escape, *k*_*E*_, that includes the rates of all steps of (abortive) initiation and elongation, plus the actual escape transition (12).

Since the rates of transcription are intimately tied to nucleotide substrate concentration, we chose a nucleotide composition that allowed us to measure the rates well by manual sampling. We used 100 μM of the 5′ initiating (“*i*” site) nucleotide, 20 μM of the 3′ initiating (“*i+1*” site) nucleotide, 100 μM of the third elongating nucleotide, and 100 μM of the appropriate 3′-dNTP. The α-^32^P labeling nucleotide corresponded to the *i+1* nucleotide so that transcripts made by single-cycle reactions could be labeled to a higher specific activity.

The time-course single-round transcription reactions mentioned previously were all performed at a template DNA to an RNAP ratio of 2:1. At this DNA-to-enzyme ratio, transcription of plasmid DNA templates, which contain other endogenous promoters, occurs exclusively from the inserted *N25-*ITS promoter. Also, under polymerase-limiting conditions, all enzyme molecules will bind to the promoter DNA and become partitioned into productive or unproductive open complexes (19). The productive fraction is responsible for the synthesis of “FL” —that is, up to the position of the 3′-deoxy analog—RNA whose finite level, during single-cycle transcription, eventually reaches a plateau. (Note: Incorporation of the 3′-deoxy analog terminates transcription, but neither the “FL” RNA nor the enzyme is released from template DNA. By “halting” the elongation complex as described and using submolar amounts of polymerase to DNA, we could establish a single-cycle condition without the addition of heparin or excess competitor DNA). In our analysis, the plateau level of FL RNA directly reflects the productive fraction of RNAP complexes formed at a promoter. By monitoring the plateau level of FL RNA synthesis from these four promoters, we can determine whether the ITS plays a role in the partitioning between productive and unproductive complexes during open complex formation.

**Figure 2:**
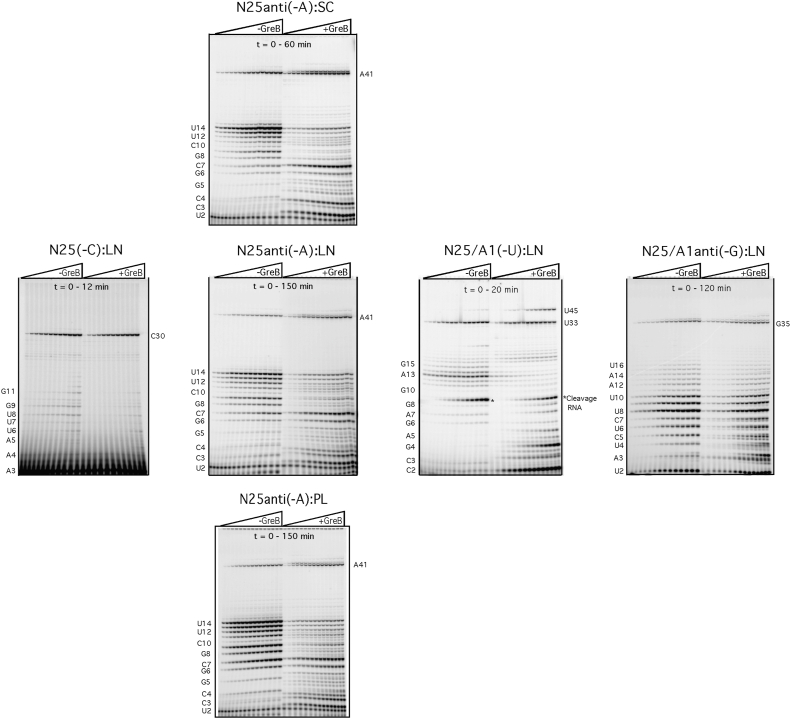
**Representative gel fractionation results of single-cycle transcription time-course experiments.** Each gel shows the parallel time-course experiments done in the absence (*left-half panel*) or presence (*right-half panel*) of GreB. *Horizontal row* shows the four promoters in the LN conformation. *Vertical column* shows, from *top* to *bottom*, the *N25anti(-A)* promoter in the SC, LN, and PL conformation, respectively. The span of the transcription time course varies from promoter to promoter because of differences in the ITS and template conformation; the span is noted on each gel. ITS, initial transcribed sequence; LN, linearized (plasmid) fragment; PL, longer PCR-derived fragment; SC, supercoiled.

Figure 2 shows typical gel fractionations of all transcripts formed in a time-dependent manner. To determine *k*_*E*_, the amount of FL RNA (y, in IQV [ImageQuant volume] units) was plotted as a function of time after the addition of triphosphates (x, in seconds or minutes) and curve-fitted to the equation y = m1 + m2^∗^(1−exp(−m3^∗^x)), which describes the exponential rise of FL RNA synthesis from zero level (at *t* = 0, all polymerases are in the form of open complexes; thus, m1 = 0) to plateau level at infinite time (m2) at a rate of *k*_*E*_ (m3). The excellent fit of our time-course data to a single exponential is indicative that the promoter escape process involves a single population of transcribing complexes (TCs) undergoing a *pseudo* first-order reaction process (*i.e*., measuring the “unimolecular” conversion of an initiated complex to an elongating complex), although it is accompanied by many steps of rapid nucleotide incorporation before and after the escape transition. Dividing *k*_*E*_ into *ln*2 yields *t*_1/2_, the half-life of escape–the span of time for half of all productive complexes to undergo the initiation–elongation transition and synthesize FL RNA. Thus, the half-life of escape is equivalent to the half-life of FL RNA synthesis.

**Figure 3:**
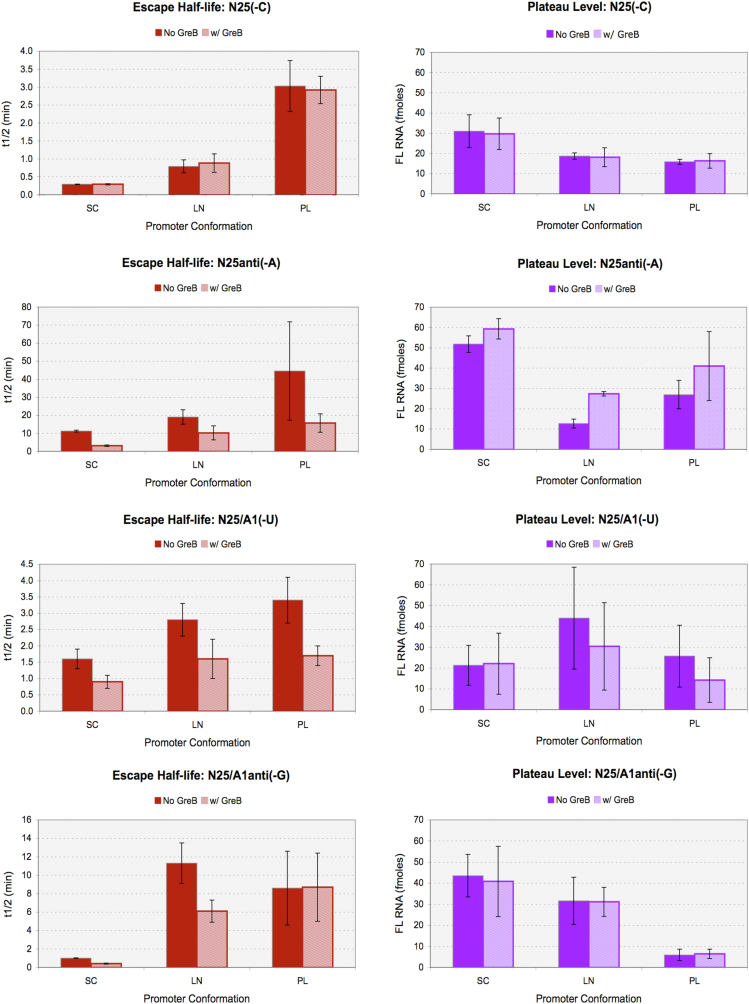
**Quantitative parameters of productive transcription from the *N25-ITS* promoters.***Right panel,* plateau level of FL RNA. The numerical data in Table 2 are plotted in *dark purple* (minus GreB) and *light purple* (plus GreB) bars. Plateau level of synthesis of FL RNA directly correlates with the productive open complexes formed at each promoter; in each time point aliquot, the maximum is 60 fmol. *Left panel,* escape half-life. The numerical data in Table 3 are plotted as *dark red* (minus GreB) and *light red* (plus GreB) *bars*. Note the difference in *y*-axis scale among the four graphs. FL, full length; ITS, initial transcribed sequence.

We performed time-course experiments three to five times for each promoter with a given template conformation and extracted the *t*_1/2_ (in minutes) and the plateau level of FL RNA (converted into fmol). The data are summarized in Tables 2 and 3 and plotted as Figure 3.

**Table 2:** Single-cycle transcription parameters versus template conformation: Plateau level of FL RNA

Promoter	N25 (-C)		N25anti (-A)		N25/A1 (-U)		N25/A1anti (-G)	
GreB	−	+	−	+	−	+	−	+
Conformation	Fmole	Fmole	Fmole	Fmole	Fmole	Fmole	Fmole	Fmole
PL	15.9 ± 1.2	16.3 ± 3.6	26.9 ± 7.0	41.0 ± 17.0	25.7 ± 14.9	14.2 ± 10.7	5.9 ± 2.7	6.4 ± 2.2
LN	18.7 ± 1.6	18.1 ± 4.7	12.7 ± 2.2	27.3 ± 1.1	44.0 ± 24.5	30.4 ± 21.0	31.6 ± 11.2	31.1 ± 6.9
SC	31.0 ± 8.1	29.7 ± 7.8	51.8 ± 4.1	59.3 ± 5.0	21.3 ± 9.6	22.1 ± 14.7	43.5 ± 10.1	40.8 ± 16.7
SC (%Prod)	∼52	∼50	∼86	∼99	∼36	∼37	∼73	∼68

All numbers are averages of three to five independent experiments ± standard deviation. SC (%Prod): the productive fraction is obtained by dividing the FL RNA level obtained with the SC template by 60 fmole and then multiplied by 100%.

**Table 3:** Single-cycle transcription parameters versus template conformation: Half-life of escape

Promoter	N25 (-C)		N25anti (-A)		N25/A1 (-U)		N25/A1anti (-G)	
GreB	−	+	−	+	−	+	−	+
Conformation	*t*_1/2_ (min)	*t*_1/2_ (min)	*t*_1/2_ (min)	*t*_1/2_ (min)	*t*_1/2_ (min)	*t*_1/2_ (min)	*t*_1/2_ (min)	*t*_1/2_ (min)
PL	3.0 ± 0.7	2.9 ± 0.4	44.5 ± 27.3	15.7 ± 5.1	3.4 ± 0.7	1.7 ± 0.3	8.6 ± 4.0	8.7 ± 3.7
LN	0.8 ± 0.2	0.9 ± 0.3	19.0 ± 4.0	10.2 ± 3.9	2.8 ± 0.5	1.6 ± 0.6	11.3 ± 2.2	6.1 ± 1.2
SC	0.3 ± 0.01	0.3 ± 0.02	11.2 ± 0.5	3.1 ± 0.4	1.6 ± 0.3	0.9 ± 0.2	1.0 ± 0.03	0.4 ± 0.06

All numbers are averages of three to five independent experiments ± standard deviation.

### Partitioning of RNAP at the open complex formation stage

RNAP partitioning during open complex formation has been seldom examined in a quantitative manner. Our reaction scheme yielded a quantitative measure of FL RNA, which, mole for mole, was synthesized by the productive complexes, thus providing us a quantitative parameter for monitoring RNAP partitioning at a promoter.

The results in Table 2 show that, depending on the ITS and template conformation, between 10% and 100% (corresponding to 6–60 fmole) of the active polymerase molecules emerged as productive open complexes. *N25/A1anti(-G)* shows the largest dependence on template conformation; the productive fraction increases from ∼10% (PL) to ∼50% (LN) and ∼68% (SC). However, this trend is not straightforward for the other promoters. For *N25anti(-A)*, when compared with the PL conformation, the productive fraction is halved in the LN but doubled in the SC conformation. *N25/A1(-U)* shows a different dependence on template conformation; compared with the PL template, its productive fraction is unchanged in the SC but doubled in the LN conformation. The productive fraction on *N25(-C)* is the same in the PL and LN conformations but doubled in the SC conformation.

The transcript cleavage factor GreB, which binds in the secondary channel of RNAP to rescue backtracked RNAs *via* cleavage, re-elongation, and escape (32), might be expected to contribute to the productive fraction of TCs. By comparing % productive complexes in the SC conformation, this was found to be the case for *N25anti(-A)* only. GreB had no effect on RNAP partitioning on *N25(-C)* and little effect on *N25/A1(-U)* and *N25/A1anti(-G)*.

While RNAP partitioning at the *N25* promoters varies idiosyncratically depending on the ITS and template conformation, we were surprised to find that *N25anti(-A)* in the SC conformation forms the highest productive fraction (at 86%). The presence of GreB seems to have converted all remaining unproductive complexes to the productive form, reaching 59.3 fmole, which is nearly the 100% level. Equally surprising is the large stimulation of productive complex formation by template supercoiling of *N25/A1anti(-G)*. In the SC conformation, the two *anti*-ITS promoters show approximately twofold higher productive fraction than their native ITS counterparts.

During RNAP partitioning to form open complexes, the ITS region remains double stranded and is anchored in the downstream binding cleft (33). It is intriguing that different ITS sequences bound in the downstream DNA-binding site can influence the extent of partitioning. More intriguing still, this sequence-dependent binding interaction is most favorable when the template is in the SC conformation. We shall address these points further in the Discussion section.

### ITS and template supercoiling significantly impact the rate of escape

As shown in Table 3, the four promoter variants that differ only in their ITS show dramatically different escape half-lives. In the PL conformation, the WT *N25(-C)* promoter has the most efficient escape, with a *t*_1/2_ of 3.0 min. *N25/A1(-U)* is nearly as efficient, showing a *t*_1/2_ of 3.4 min, whereas *N25/A1anti(-G)* achieves escape with a half-life of 8.6 min. *N25anti(-A)* is extremely slow at escape and shows an escape half-life of ∼45 min. Comparing the escape kinetics of these promoter variants, it is clear that the *antiDSR* ITS has introduced a severe rate impairment on escape.

For each promoter, the escape half-life is further dependent on template conformation (Fig. 3, *left panel*). In general, the longer the template DNA, the faster the rate of escape. Escape becomes the most facile when template DNA is SC. In the SC conformation, the *t*_1/2_ of escape for *N25(-C)* is shortened to 0.3 min; for *N25anti(-A)*, to 11.2 min; for *N25/A1(-U)*, to 1.6 min; and for *N25/A1anti(-G)*, to 1.0 min. In this regard, *N25/A1anti(-G)* derives the most stimulation from supercoiling (∼20-fold), *N25/A1(-U)* gains the least (only 2.5-fold); and *N25(-C)* and *N25anti(-A)* each gain 7- and 4-fold, respectively.

GreB further enhances the rate of escape on non-native ITS variants of *N25* promoter, as shown in Table 3 and Figure 3, and reduces their *t*_1/2_ by 2- to 3-fold but has no effect on the escape rate of *N25(-C)*. Overall, GreB appears to have hastened escape more substantially from the *anti*-ITS promoters than from their respective native counterparts. Likely this is due to the higher level of abortive initiation undergone by these promoters during escape (Fig. 2). The inclusion of GreB, therefore, allows us to tease out the two components—abortive initiation and productive escape—that make up the rate of escape measurement. In the absence of GreB, a single population of initial transcribing complexes (ITCs) undergoes stochastic rounds of abortive initiation before escape; the population as a whole is slow escaping. When present, GreB rescues the backtracked complexes and facilitates the passage of RNAP through the early steps to the escape stage, thus converting the ITCs into a fast-escaping population. That *N25(-C)* shows no difference between ±GreB conditions is indicative that the *N25* promoter has evolved an ITS that is optimally suited for the promoter escape challenge. The single population of *N25*ITCs is fast escaping with or without GreB. Its productive synthesis, nevertheless, is accompanied by “obligatory” abortive RNA release, albeit at a low level (Fig. 2).

The T5 *N25* promoter escape rate was previously measured using a molecular beacon assay with a fluorescently labeled σ^70^ subunit on a PCR fragment template (34). The escape process was found to follow single-exponential kinetics. Petushkov *et al.* (34) found a *t*_1/2_ of 51.5 ± 4.5 s for T5 *N25* and 51.8 ± 7.9 s for T5 *N25cons*, suggesting that the WT *N25* promoter sequence is essentially “consensus” in nature. The *t*_1/2_ we obtained for T5 *N25* in the PL format is 180 ± 42 s. Our rate measurement compares reasonably well with the above when we take into account the differences in reaction conditions and assay design. Where our two studies differ is the effect of GreB on escape rate. Whereas we saw no effect of GreB on the WT *N25* promoter (at GreB:RNAP ratio of 10:1), Petushkov *et al.* (34) found a nearly doubled rate enhancement on escape, with a *t*_1/2_ of 27.5 ± 1.6 s (albeit at a GreB:RNAP ratio of 250:1).

### Kinetics of abortive RNA synthesis pinpoints a barrier in the unproductive ITC

**Figure 4:**
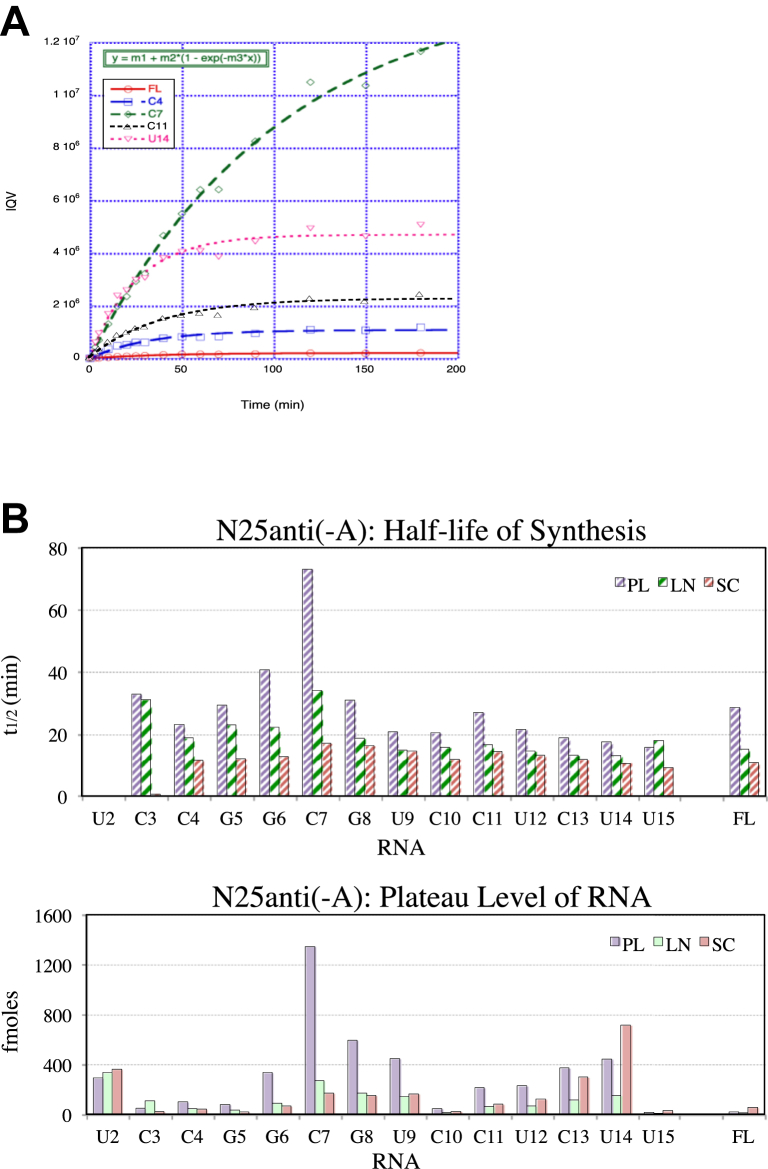
**Abortive and productive RNA synthesis kinetics of *N25anti(-A*) promoter.***A,* kaleidagraph plot of the time course of formation of several abortive RNAs: C4 in *blue*, C7 in *green*, C11 in *black*, and U14 in *pink*; and FL RNA in *red*. *B, top panel,* plot of half-life of synthesis of abortive and FL RNAs from Table 4. *Bottom panel,* plot of plateau level of synthesis of abortive and FL RNAs from Table 4. FL, full length.

*N25anti(-A)* in the PL conformation is a highly abortive promoter. Although abortive RNAs are known to derive from both the productive and unproductive ITCs (19), it has not been possible, without knowing the FL RNA synthesis kinetics of the productive ITCs, to resolve the two types of complexes and study their abortive RNA formation kinetics individually. However, the transcriptional time-course results displayed by PAGE in this study allow quantitation of the individual abortive RNAs. By analyzing the abortive RNA formation kinetics, we show that all abortive RNA syntheses also follow a single exponential increase like the FL RNA (Fig. 4*A*). The existence of a synthesis plateau allows us to extract *t*_1/2_ and the plateau level of synthesis for all abortive RNA species (Table 4; Fig. 4*B*). In comparing the *t*_1/2_ values, we reasoned that those abortive RNAs with similar or shorter *t*_1/2_ than FL RNA are likely produced by the productive ITCs; these included abortive RNAs longer than G8 in the PL template (which formed 19 fmole [∼32%] of productive open complexes that escaped with a *t*_1/2_ of 28 min). Abortive RNAs G8 and shorter are likely produced by both the productive and unproductive ITCs. Among the latter, we note the anomalously high level of C7 RNA (1346 fmole) synthesized with a half-life of 73 min. These two measurements suggest the existence of an ITC that undergoes abortive initiation reiteratively to synthesize the C7 RNA. At this length, for normal transcription to continue, the nascent RNA should have encountered the σ3.2 finger blocking the RNA exit channel and begun to displace it out of the channel (35, 36). Functionally, the C7-producing ITC appears to be stuck in a conformation unable to accommodate a nascent RNA beyond the 6-nt stage; it fits the description of an unproductive ITC.

**Table 4:** Kinetic parameters of abortive and productive transcription on *N25anti(-A)* promoter

Quantitative parameter	Plateau level of RNA			Half-life of synthesis		
Conformations	SC	LN	PL	SC	LN	PL
RNA	(fmol)	(fmol)	(fmol)	(min)	(min)	(min)
U2	360	336	292			
C3	21	107	49	1	31	33
C4	41	46	101	11	19	23
G5	20	35	77	12	23	29
G6	68	87	336	13	22	41
C7	168	**268**	**1346**	**17**	**34**	**73**
G8	153	168	592	16	19	31
U9	164	144	446	14	15	21
C10	22	15	43	12	16	20
C11	82	61	212	14	17	27
U12	121	68	229	13	14	21
C13	298	115	373	12	13	19
U14	**713**	149	442	10	13	18
U15	29	6	16	9	18	16
**FL**	**56**	**11**	**19**	**11**	**15**	**28**
% Productive:	∼93%	∼18%	∼32%			

The highest number in each abortive RNA column is *bolded* and, as well, the values for FL RNA. % Productive: the productive open complex fraction associated with each template conformation.

The C7 block is greatly diminished in the SC template because most polymerases (56 fmole, or ∼93%) are now in the productive conformation. While escape occurs at +15, there appears now a new barrier to escape at the U14 position. This result is consistent with ITC14 being on the cusp of escape, straddling between the pretranslocated and post-translocated states. Pretranslocation, the ITC14 tends to backtrack and release the U14 RNA. Post-translocation, the enzyme active center is positioned at +15, now the escape maneuvers can occur. (As detailed later in the Discussion section, the escape maneuvers include three steps: promoter release, upstream bubble collapse, and downstream elongation bubble formation.)

We analyzed the abortive RNA synthesis kinetics for the other three *N25* promoter variants but failed to find another example of an unproductive ITC, suggesting that the synthesis and abortive release of C7 RNA might be sequence specific. The NT dinucleotide sequence at +7/+8 on *N25anti(-A)* is CG, which corresponds to the YG dinucleotide sequence motif found for all known pause sites (37). The likelihood that the abortive release of C7 RNA is preceded by a promoter-proximal pause cannot be ruled out (38, 39).

To summarize the aforementioned, we have identified a *bona fide* unproductive ITC species on the *N25anti(-A)* PL template that is unable to transcribe past the 7-nt stage, possibly because this nascent RNA cannot be accommodated in the RNA exit channel (35, 38). This ITC species pauses temporarily before backtracking occurs to release the 7-nt RNA. This defect appears to be remedied in the SC template since nearly all ITCs are now in the productive conformation, which displays a new escape barrier at U14.

## Discussion

In this study, we quantitatively measured two parameters that govern the productive synthesis from the escape rate–limited *N25*-based promoters. The following are the parameters: (1) the productive fraction (resulting from RNAP partitioning into productive *versus* unproductive open complexes), and (2), *k*_*E*_, a composite rate constant of escape expressed as *t*_1/2_, the half-life of escape. Our results show that both parameters are highly affected by the ITS and template supercoiling (Tables 2 and 3). To understand their respective roles in the functioning of an RNAP, we draw on the extensive information known about the open complex formation process on the λ *P*_*R*_ promoter (33). Our kinetic data are also considered in the context of many recent high-resolution determinations of (scrunched) open complex and ITC structures (20, 21, 22, 23, 40, 41, 42). The result of this analysis is the proposal of a step-by-step process of transcription initiation–promoter escape for the T5 *N25-ITS* promoters that is consistent with our kinetic measurements and the roles of ITS and template supercoiling in the promoter escape mechanism.

### Open complex formation process is best studied with λ P_R_

The T5 *N25* promoter has the highest *k*_on_ value when interacting with RNAP and the longest open complex half-life among a group of strong promoters investigated by Brunner and Bujard (11). However, not much is known regarding its open complex formation process, except for the fact that biochemically it forms two types of open complexes—productive and unproductive—like other strong Eσ^70^ promoters, for example, T7 *A1*, *lac*CONS, and λ *P*_*R*_ (17, 19, 33, 43, 44). Of the aforementioned promoters, only λ *P*_*R*_ has been investigated comprehensively for its open complex formation process (33, 44, 45). The multistep process occurs sequentially from RPc to the first unstable and short-lived open complex I_2_ (46), which then quickly converts into long-lived open complexes I_3_ and RPo (33, 45). The I_2_ to I_3_ conversion involves the folding/assembly/binding of the RNAP β′ clamp/β lobe on proximal downstream duplex DNA from +3 to +10, whereas the I_3_ to RPo conversion recruits the β′ jaw and nearby mobile elements to assemble onto the distal downstream DNA from +11 to +20 (45). The downstream duplex DNA from +3 to +20 constitutes the ITS region of a promoter. Several other studies have also directly shown the importance of downstream duplex DNA binding in the formation of complexes competent at transcription initiation (47, 48).

The series of structural investigations on λ *P*_*R*_ culminated in the resolution of its open complex structures by single-particle cryo-EM methodology (22). This study revealed two classes of structures with sharp differences in the melted bubble region. Class I complex contained a fully resolved template (T) and nontemplate (NT) single strands with the nucleotides from −11 to +2 arranged in a well-ordered and stacked conformation, further stabilized by numerous interactions with RNAP residues. The NT strand was especially well accommodated with four flipped bases (A_−11_, T_−7_, G_−5_, and N_+2_), each ensconced in its own hydrophobic binding pocket on the σ_2_, σ_2_, σ_1.2,_ and β subunits, respectively (49, 50, 51). By contrast, in the class II complex, all single-stranded nucleotides—except for the NT nucleotides −11 to -5—were unresolved and presumably relatively free and devoid of structured regions.

The two classes of λ *P*_*R*_ open complex further differed in the strength of upstream and downstream duplex interactions. The class I complex showed a well-resolved proximal αCTD–UP DNA interaction upstream and two turns of downstream duplex DNA in its binding site, whereas the class II complex showed an unresolved upstream αCTD–UP DNA interaction and just one turn of downstream duplex DNA binding. The well-ordered class I complex structure was deemed highly stable and “rigid,” and therefore, likely represented the unproductive open complex. The class II complex was flexible and “dynamic,” thus deemed to represent the productive open complex.

Of the two long-lived open complexes, I_3_ and RPo, Plaskon *et al.* (52) showed that I_3_ was the productive complex (with the class II structure), and it was formed before the unproductive RPo (with the class I structure). The I_3_ to RPo conversion involves further β′ clamp closure (33, 45), which extends and strengthens the downstream duplex DNA contacts, thereby leading to restructuring of the polymerase upstream contacts, including the transcription bubble, thus resulting in an RPo that is highly stable and rigid (22). The sequential formation *first* of the productive complex (I_3_) followed by the unproductive complex (RPo) shows how a branched pathway of transcription initiation can arise.

### Inferring an open complex structure for the T5 N25 promoter

**Figure 5:**
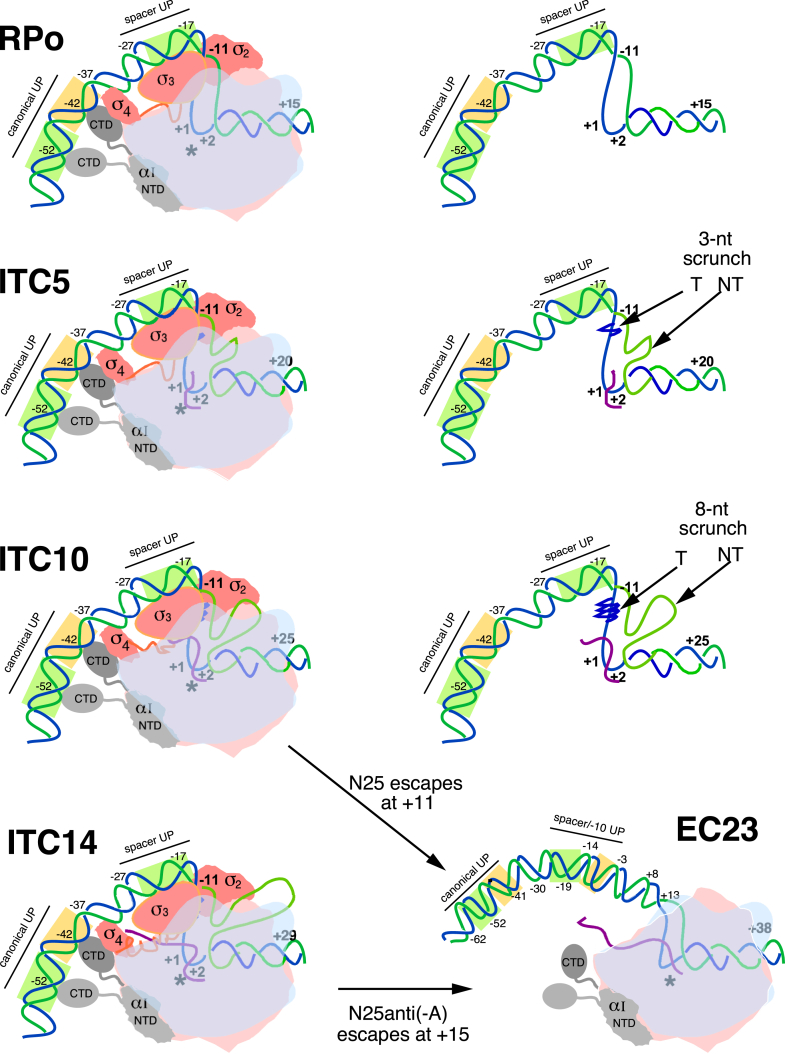
**T5 *N25* promoter transcription complexes.** Shown are the schematic renderings of an RPo, an ITC5, an ITC10, an ITC14, and an EC at +23; and in parallel, the DNA–RNA skeleton of RPo, ITC5, and ITC10. *N25(-C)* initial transcription involves RPo, ITC5, and ITC10; escape occurs at +11 and EC11 ensues as a result. *N25anti(-A)* initial transcription involves RPo, ITC5, ITC10, and ITC14; escape occurs at +15, and EC15 ensues as a result. During initial transcription, the outward appearance of the RPo and ITCs are not too different; they are all similarly anchored in the upstream DNA region and differ slightly in the downstream boundary. What differentiates them are the scrunching-induced transcription bubble distortions and the presence of a nascent RNA. EC11 (not shown), EC15 (not shown), and EC23 all have similar structural features of an 11-bp elongation bubble and unbent upstream DNA. Here, EC23 is shown to have released the sigma subunit. Components of the transcription complexes by color are as follows: β′ subunit, *pink*; β subunit, *blue* (made 30% transparent for viewing the nucleic acids inside the enzyme active site cleft); α subunits, *light and dark gray*; σ subunit, *red*; DNA T-strand, *dark blue*; NT-strand, *green*; and nascent RNA, *purple*. The *asterisk* denotes the catalytic center, which is located at the *bottom* of the active site cleft situated between +1 (the “*i*” site) and +2 (the “*i + 1*” site) positions. The two UP element subregions are shown as *gold* (proximal) and *light green* (distal) *rectangles*. The T-strand scrunch, localized to the −9 to −11 region, is represented by one or more *dark blue zigzag symbols*; the NT-strand scrunch extrudes out of the enzyme downstream of the −4 position. ITC, initial transcribing complex; NT, nontemplate; RPo, open complex; UP, upstream activating element.

T5 *N25* and λ *P*_*R*_ share a high degree of homology in their promoter architectures. The *N25* core promoter sequence (from −60 to −1) shows a near consensus (-35 element [TTGctt]/17-bp spacer/-10 element [TATAAT]) arrangement resembling that of λ *P*_*R*_ (-35 element [TTGACt]/17-bp spacer/-10 element [gATAAT]). Both promoters also contain a canonical UP element centered at −49 showing ∼66% homology to the full UP element consensus (53, 54). They also contain a 6-bp DIS with G_−5_ residue, which binds specifically in a pocket on σ1.2 to confer a long open complex half-life (22, 50). Interestingly, the *N25* promoter contains an additional UP element in the spacer region centered at −19 showing 91% homology to the distal UP element consensus (54). This AT-rich spacer DNA was shown to be utilized by αCTD subunits during the escape transition on an *N25*-ITS promoter variant *DG203* (54). However, the spacer UP element is not in contact with the α subunits at the open complex stage. Rather, this AT-rich spacer sequence, which closely resembles the *fullcon* spacer sequence (54, 55, 56), can bend and adopt a trajectory that enables better anchoring of the upstream duplex DNA on the surface of RNAP at the σ_3_ region (56, 57). The aforementioned information gives rise to an *N25* promoter open complex structure of comparable, if not higher, stability than λ *P*_*R*_. A schematic drawing of a T5 *N25* promoter open complex structure is shown in Figure 5. Last, we propose by analogy to λ *P*_*R*_ also that the structural differences between productive and unproductive *N25-ITS* open complexes result from further tightening of the downstream duplex DNA binding, converting a fraction of the initially productive open complexes to unproductive ones.

### Role of the ITS in RNAP partitioning during open complex formation

The four *N25-ITS* variants examined here are identical in upstream promoter sequence from −60 to −1, but they differ in the downstream sequence from +1 to +20, that is, the ITS. Our results show that all four variants form productive and unproductive complexes during the later steps of open complex formation (Table 2). The fraction of productive complexes varies with the ITS and with the length of the template DNA. Within each variant, the productive fraction increases sharply with template supercoiling. The binding of a duplex ITS element to the downstream DNA-binding site at the RNAP β′ jaw region is known to activate open complexes for transcription initiation (33, 45, 48). However, the four ITSs giving rise to rather different extents of productive complexes is unexpected. This result indicates that there is a sequence preference conferred by the downstream DNA-binding site on the ITS. Sequence characteristics of the proximal duplex ITS DNA (from +3 to +13)—the region stabilized in the class II productive open complex structure (22)—show AT richness for *N25(-C)*: 8/11 bp and *N25/A1anti(-G)*: 7/11 bp but GC richness for *N25anti(-A)*: 9/11 bp and *N25/A1(-U)*: 7/11 bp. However, there is no clear correlation of AT/GC richness with high/low productive fraction. Interestingly, the two variants with the highest productive fraction—*N25ant(-A)* and *N25/A1anti(-G)* in their SC conformation—show CT richness in the nontemplate strand of their proximal ITS. Whether this sequence preference proves relevant will require further investigation of a larger sample size of promoter-ITS variants.

Within each promoter variant, the SC template exceeds the linear (PL and LN) templates in fostering the productive open complex formation. How does supercoiling support the formation of productive complexes? One possibility might be that the duplex ITS sequence, being an integral part of an SC plasmid DNA molecule, is structurally more constrained, and, therefore, better anchored in the downstream DNA-binding site. Thus bound, the complex can resist further encroachment by the β′ jaw closure to form the unproductive complex (45). By contrast, this conversion is readily achieved with relaxed (and likely flexible) duplex DNA in the downstream binding site.

### Rate of promoter escape varies with the ITS and template conformation

Our kinetic measurements reveal a sizeable role of the ITS and template conformation in modulating the rate of escape (Table 3). While the *N25* promoter with the native ITS is the most facile at escape, the non-native ITSs affect the rate of escape in varying ways such that, in general, they pose an impediment to escape. Within each ITS variant, the rate of escape is further dependent on the upstream duplex DNA length and conformation, with template supercoiling providing the largest boost to escape. Below, by focusing on the large differences in rate and abortive transcription pattern observed with *N25(-C) versus N25anti(-A)* in the SC format (Fig. 2 and Table 3), we can deduce their respective roles by summarizing a step-by-step process of transcription initiation–promoter escape. This model is based on current structural understandings of various TCs (20, 21, 22, 23, 40) and incorporates the kinetic findings on *lac*CONS, λ *P*_*R*_, and T7 *A1* promoters (52, 58, 59).

### A step-by-step model of transcription initiation to promoter escape

#### Step 1: Transcription initiation proceeds *via* scrunching to generate a stressed intermediate at each step

RNAP translocates *via* scrunching (60, 61). Upon initiation from a stably held promoter open complex, the ITS increasingly becomes unwound, and the new single strands are drawn into the enzyme active site cleft. Scrunching increases the number of nucleotides in the transcription bubble but compacts it at the same time, generating disruption in the protein–DNA contacts as well as backbone distortion in the single strands. This creates a high-energy stressed intermediate, which corresponds to the transition-state complex between the pre- and post-translocated forms of an ITC. A high-energy stressed intermediate is generated by scrunching translocation after each step of nucleotide incorporation (62).

#### Step 2a. Scrunching of the ITS determines the abortive transcription program in a sequence-specific manner

At each step, the high-energy stressed intermediate undergoes some stress relief by reaching a local energy minimum before the next nucleotide incorporation can occur. Energy minimization *via* reversible translocation (29, 52) can occur either by processive unscrunching (backtracking), which can dissipate the stress and lead to abortive release of the nascent RNA, or by forming new enzyme–DNA contacts to accommodate the distorted DNA. “Accommodation” yields the next ITC with a new energy minimum and enables the next nucleotide incorporation to grow the nascent RNA. The relative ease of stress dissipation *versus* stress accommodation determines the abortive potential at each of the initial steps.

Comparing the abortive profile of *N25(-C) versus N25anti(-A)* (Fig. 2), it is clear that the AT-rich native ITS is well accommodated by the RNAP active site cleft, but the GC-rich *anti*-ITS is not well accommodated. The reason for the former is the identical sequence composition—9 AT and 2 GC bps—of *N25* ITS (from +1 to +11) to the open complex bubble strands (from −11 to −1); the latter is responsible for the high *k*_on_ and long *t*_1/2_ for T5 *N25* RPo. As the enzyme draws in the ITS region, it is not surprising to find the AT-rich ITS of *N25(-C*) making its way through the initial steps to reach promoter escape at +11 with a rapid rate (*t*_1/2_ of 0.3 min) while releasing just a trace level of abortive RNAs (2–10 nt).

The passage of the GC-rich ITS of *N25anti(-A)* through the enzyme active site cleft clearly requires difficult “accommodation,” as evidenced by the high level of abortive RNA release throughout initial transcription. In addition, the higher reannealing potential of the GC-rich ITS single strands favors stress dissipation by backtracking. These factors render the polymerase “trapped” at the slow steps of initial transcription—that is, reverse translocation of the RNAP active center and dissociation of the abortive RNA (58, 63)—and can account for the greatly compromised rate of promoter escape (*t*_1/2_ of 11 min). This observation also clarifies the large rate enhancement obtained in the presence of GreB (*t*_1/2_ of 3 min). By binding in the secondary channel, GreB actively circumvents the slow steps by suppressing backtracking and rescuing the backtracked RNAs from abortive release *via* transcript cleavage followed by re-elongation (64, 65, 66).

#### Step 2b: Cumulative scrunching–induced stress is harbored on the template strand

As the nascent RNA grows, the scrunched DNA also becomes cumulatively longer. How the scrunched DNA is accommodated in an ITC is now well documented from protein–DNA crosslinking-mapping analysis (20, 40) and structural resolution of (scrunched) open and ITCs (21, 22). Two features of the NT strand render it relatively stress free during initial transcription: (1) the persistent binding of A_−11_, T_−7_, and G_−5_ residues in their respective binding pockets on σ2, σ2, and σ1.2 and (2) the ability to extrude distorted DNA (3 nt and longer) to the enzyme exterior downstream of the −4 (λ *P*_*R*_)/-5 (T7 *A1*) position. Empirically, this was accidentally shown to be the case on the T5 *N25* promoter, where abortive transcription of the +8 RNA is unaffected by stress-relieving disruptions (*i.e.*, mismatches, nicks, and gaps) introduced in the NT strand near the downstream end of the transcription bubble (67). Similarly, manipulating the NT strand to relieve the scrunching stress in the λ *P*_*R*_ transcription bubble failed to show any changes in the promoter escape properties (68).

Stress accommodation on the T-strand is quite different. The nascent RNA and T-strand scrunch simultaneously become 1-nt longer after each nucleotide incorporation. Both elements—when they are longer—can disturb the structural integrity of a TC. At 5-nt long, the nascent RNA begins to make contact with the σ3.2 fingertip that blocks the RNA exit channel. As synthesis continues, the longer RNA is involved in displacing the σ3.2 finger/loop out of the RNA exit channel (35, 36). Meanwhile, the T-strand scrunch is localized to the −11 to −9 positions near the upstream double-stranded/single-stranded junctions of the bubble; importantly, this arrangement leaves the length of T-strand sequence from −8 to the active site free to form an RNA–DNA hybrid during the initial transcription steps (22). Crystal structure and protein crosslinking evidence both indicate that a T-strand bulge of 3 nt is not extruded but is well contained in an internal protein chamber capped by the globular domain of σ3 (20, 21). A schematic drawing of such an ITC5 (with a 3-nt scrunch) is shown in Figure 5. With a 5-nt scrunch, there emerges an outward shift of the σ3 domain, which potentially can move away the σ3.2 blockage of the RNA exit channel to facilitate nascent RNA synthesis. This shift can also interfere with the β-flap/σ4.2/-35 DNA interaction anchoring the upstream duplex DNA (21). Structures of Eσ^70^ ITCs with longer scrunches are currently unavailable. However, the structures of an entire series of eukaryotic pol II-super core promoter TCs (TC2–TC17) were solved recently (23). The findings from this study greatly clarify the structural rearrangements on TCs undergoing the initiation–elongation transition (to be discussed in *Step 3* later).

#### Step 2c: Scrunching provides a driving force for promoter escape

T5 *N25* and λ *P*_*R*_ both transcribe to synthesize a range of abortive RNAs 2 to 10 nt in length before undergoing promoter escape at +11. To reach the initiation–elongation transition phase, an ITC10 must undergo scrunching-translocation to the +11 position. This intermediate complex, which is on the cusp of escape, would harbor an (n−2) or 9-nt scrunch (60) and accrue ∼9 to 18 kcal/mol of stress energy (*i.e.*, based on an estimate of ∼1–2 kcal/mol per scrunched nucleotide) (40, 63, 69, 70). When deployed, this amount of stress energy is sufficient to disrupt the RNAP–sigma interactions (∼13 kcal/mol) and the sigma–promoter DNA interactions (∼7–9 kcal/mol) (71), leading to promoter escape and the start of the elongation phase. On T7 *A1*, the longest abortive RNA is the 7-mer, and escape occurs at +8. The *A1* ITC at +8 harbors a 7-nt scrunch (6 from nascent RNA synthesis plus 1 from its 7-bp DIS (22)), which generates ∼7 to 14 kcal/mol of stress energy that is available for promoter escape. Its escape transition is reached earlier than with T5 *N25* or λ *P*_*R*_ because of the formation of a less stable open complex with a shorter half-life (*k*_on_: 1.6 × 10^8^ M^−1^ s^−1^; *t*_1/2_: 12.5 min; *K*_eq_ = 1.7 × 10^11^ M^−1^) (11, 59). In keeping with this trend, we show that on λ *P*_*L*_ (*k*_on_: 0.1 × 10^8^ M^−1^ s^−1^; *t*_1/2_: 17 min; *K*_eq_ = 1.5 × 10^10^ M^−1^) the longest abortive RNA is the 6-mer; thus, escape occurs at the +7 position (11, 25, 72). A λ *P*_*L*_ ITC7 on the cusp of escape would harbor a 5-nt scrunch providing ∼5 to 10 kcal/mol of stress energy to bring about escape. This correlation of the position of escape with open complex stability—that is, the more stable an open complex, the longer its abortive transcription program—is consistent with the idea that scrunching-induced stress energy provides the driving force for promoter escape (60). This correlation holds well with the aforementioned “natural” promoters but, as we shall see later, breaks down with the *N25-ITS* variants, especially *N25anti(-A).*

#### Step 3: A structural perspective of promoter escape at the initiation–elongation transition

The abortive initiation–promoter escape process has been captured in cryo-EM structures of eukaryotic RNAP pol II- super core promoter transcription complexes (TCn), from initiation (TC2–TC9) to early elongation (TC10–TC17) (23). This study showed that the initial RNA synthesis from 2 to 9 nt is accompanied by transcription bubble expansion from 14 to 21 nt. Escape occurs at the TC9–TC10 juncture, where large conformational changes coincide with the dissociation of the general transcription factors, TFII-B, -D, -E, -F, and -H—the eukaryotic equivalent of the bacterial sigma subunit—allowing the upstream promoter DNA to unbend. The unbending enables the 21-nt transcription bubble in TC9 to collapse to an 11-nt elongation bubble containing a 10-bp RNA–DNA hybrid in TC10 (73). The elongation bubble remains fixed at 11 nt throughout early elongation from TC10 to TC17, similar to the situation with *E. coli* RNAP elongation complexes (74). Detailed analysis led these authors to conclude: (1), the pol II initiation–elongation transition is the result of cumulative scrunching–induced stress in the T-strand DNA; and (2), the initiation–elongation transition at the TC9–TC10 juncture involves general transcription factor dissociation, bubble collapse, and promoter escape. Most importantly, this study showed that, upon escape, the upstream bubble collapses to leave open an 11-nt elongation bubble. This finding, although shown with a eukaryotic pol II complex, can be appropriated for explaining the escape transition in bacteria (75) and is crucial for our comprehension of the vastly different kinetics and escape pattern of *N25(-C) versus N25anti(-A).*

#### Step 3a: Dissecting the N25 promoter escape process into three steps

The three steps are promoter release, upstream bubble collapse, and downstream elongation bubble formation/promoter escape. Promoter release is brought about by scrunching–translocation of the previous ITC and frees the upstream duplex DNA from its 90^o^-bending constraint, forming an expanded bubble intermediate that is on the verge of escape. Once unbent, rewinding of the upstream portion of the expanded bubble—especially aided by template supercoiling (see *step 3b* later)—can occur, and results in the formation of the first elongation complex containing an 11-nt bubble.

**Figure 6:**
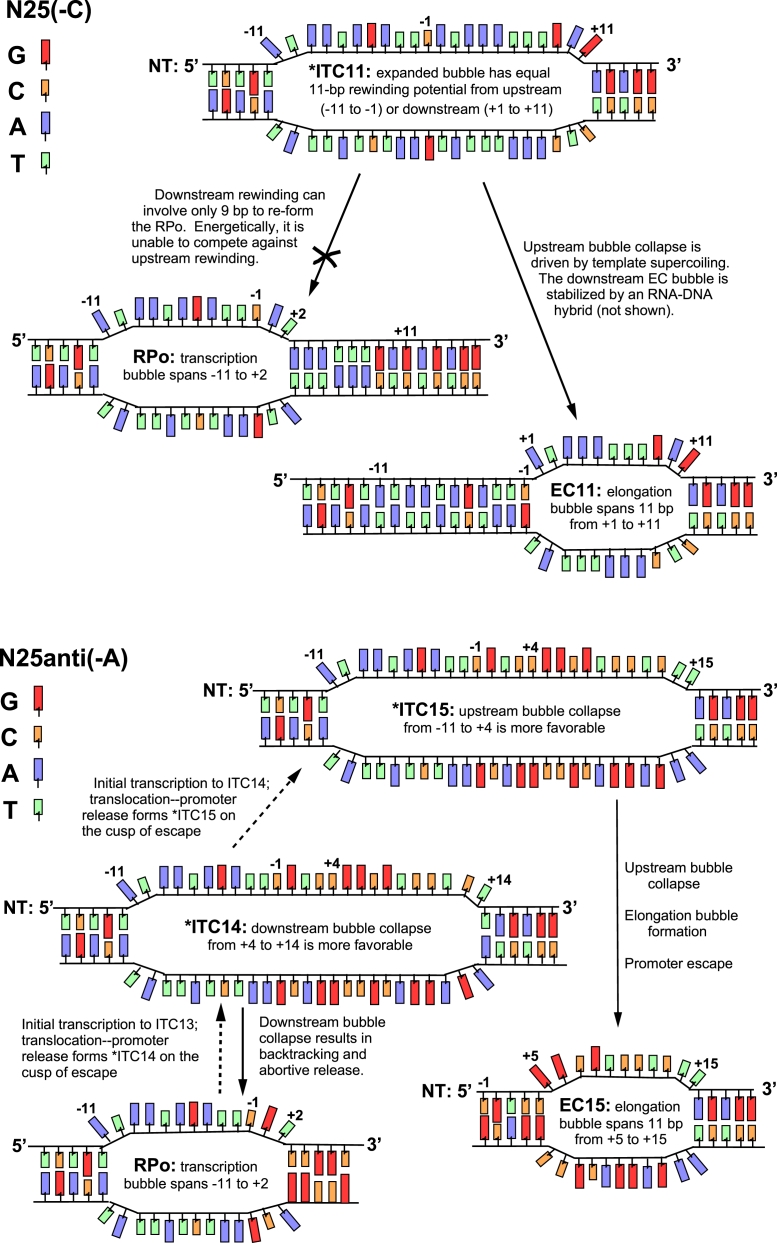
**Upstream *versus* downstream rewinding in an expanded bubble intermediate.** The DNA in the promoter complexes is shown in four colors—G, *red*; C, *orange*; A, *blue*; and T, *green*—to better convey the sequence characteristic differences among the AT-rich open complex bubble region (*blue/green*), the AT-rich *N25(-C)* native ITS (*blue/green*), and the GC-rich *N25anti(-A)* ITS (*red/orange*). Once in the bubble conformation, the GC-rich region rewinds more readily than the AT-rich stretches. Not shown are the nascent RNAs associated with the ITCs and ECs. *Top panel,* N25(-C). Initial transcription proceeds to ITC10, which, upon scrunching-translocation followed by promoter release, forms an expanded bubble intermediate designated as ^∗^ITC11. ^∗^ITC11 is distinguishable from ITC11, which has not undergone promoter release. While its catalytic center is positioned at +11, incorporation of the 11th nucleotide cannot occur until the expanded bubble intermediate undergoes upstream bubble collapse to form an 11-bp elongation bubble. As shown, ^∗^ITC11 can only undergo upstream bubble collapse to form EC11. *Bottom panel,* N25anti(-A). Initial transcription can lead to expanded bubble intermediates at the +11, +12, +13, and +14 positions, but none of these can complete the escape transition (Fig. 7). Escape only occurs with ^∗^ITC15. Here, we show the two expanded bubble intermediates, ^∗^ITC14 and ^∗^ITC15. In ^∗^ITC14, the downstream bubble collapse (from +4 to +14) is energetically more favorable than upstream rewinding (from −11 to +3); backtracking followed by abortive release results in reforming the RPo. In ^∗^ITC15 (which arises from an independent round of initial transcription), upstream bubble collapse (from −11 to +4) is energetically more favorable than downstream rewinding (from +5 to +15); EC15 is formed as a result. ITC, initial transcribing complex.

*N25(-C)*, which undergoes the promoter escape transition at +11, first forms (from ITC10 undergoing scrunching–translocation followed by promoter release) an expanded bubble intermediate spanning 22 nt (from −11 to +11). We designate this intermediate as ^∗^ITC11; its catalytic center is positioned at +11, but no nucleotide incorporation can occur until the upstream bubble collapses to form an 11-nt elongation bubble downstream. On *N25(-C)*, the upstream half of the expanded bubble (from −11 to −1) rewinds preferentially to leave open the downstream half (from +1 to +11) as an 11-nt elongation bubble (Fig. 6, *top panel*). When escape is complete, transcription elongation can start from the +11 position to produce FL RNA. On *N25(-C)*, the three steps are tightly linked and proceed with rapid kinetics.

On *N25anti(-A)*, however, all three steps are distinctly different because of its highly GC-rich ITS. While it is expected to undergo promoter release at the same position as *N25(-C)* (because of identical open complex stability for these two variants (11)), release/escape only occurs at +15.

**Figure 7:**
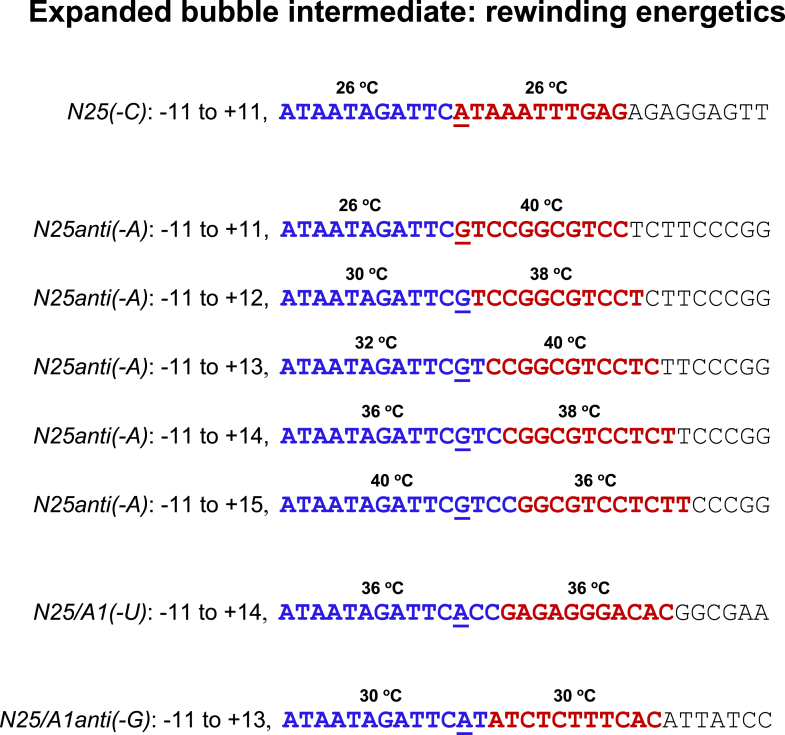
**Expanded bubble intermediate: rewinding energetics.** An expanded bubble intermediate can undergo either upstream or downstream bubble collapse. Which one prevails is dependent on their relative energetics of rewinding. The NT-strand sequence from −11 to +20 is shown for each promoter. The highlighted sections (*blue* and *red*) correspond to the expanded transcription bubble just before escape. Upstream bubble collapse (*blue*) is successful only when the energy released during rewinding is sufficient to keep the downstream 11-bp bubble (*red*) open as the first elongation bubble. The *T*_m_ of melting for each highlighted section is calculated using the formula: *T*_m_ = 2 °C (#AT) + 4 °C (#GC). For *N25/A1(-U)* and *N25/A1anti(-G)*, their precise position of escape could not be obtained from the abortive transcription pattern seen in gels (Fig. 2) but is predicted to occur at C14 and C13, respectively, based on *T*_m_ calculation. NT, nontemplate.

The sharp differences in their position of escape prompted the following proposal. That, at the escape juncture, which portion of the expanded bubble collapses depends on the relative energetics of rewinding the upstream region of the expanded bubble *versus* keeping open a downstream 11 bp (in the melted ITS region) as the first elongation bubble. The variable part of these energetics attributable to DNA–DNA interactions can be approximated by *T*_m_ calculation of oligonucleotides based on sequence composition (76); the result is shown in Figure 7. (Here, we are justified in only considering the DNA–DNA interactions for the following reasons: (1) the *T*_m_s are relevant if the DNA–protein interactions are similar in each case; (2) at the escape juncture, the DNA–protein interactions are either in flux or momentarily absent because of the large conformational changes occurring on the enzyme–protein complex; and (3), the RNA–DNA hybrid associated with the downstream bubble region appears not to convey stabilization against reannealing when backtracking, and abortive release can occur with high efficiency, especially at the +11 to +14 positions on *N25anti(-A)* (Fig. 2)).

As shown in Figure 7, upstream bubble collapse for *N25anti(-A)* is energetically untenable with promoter release occurring at the +11, +12, +13, or +14 position. It becomes favorable only at +15 when the upstream bubble from −11 to +4 collapses, releasing more energy than necessary to keep open the downstream 11-bp elongation bubble spanning +5 to +15. (We illustrate this in Figure 6, *bottom panel*, with ^∗^ITC14 and ^∗^ITC15. ^∗^ITC14 undergoes backtracking and abortive release from energetically more favorable downstream bubble collapse. ^∗^ITC15 undergoes escape because of the energetically more favorable upstream bubble collapse). Thus, on *N25anti(-A)*, there exists a rate-limiting (slow) step at upstream bubble rewinding. (This is because, of all the expanded bubble intermediates—^∗^ITC11, ^∗^ITC12, ^∗^ITC13, ^∗^ITC14, and ^∗^ITC15—only ^∗^ITC15 can undergo the escape transition). Where the *first* elongation bubble can form is dependent on the sequence composition of the ITS. Escape occurs only when all three integrally linked steps can be accomplished successfully.

#### Promoter release *versus* sigma release

At the promoter escape transition, we surmise that only the σ3/spacer DNA, σ4.2/−35 DNA, and the σ_3_–σ_4_/core polymerase contacts are broken to release the upstream duplex DNA, allowing it to unbend. The strong σ_2_/core and σ_2_/−10 DNA interactions likely remain intact (77). The latter speculation is based on the high level of abortive initiation undergone by *N25anti(-A)* promoter, especially at the +11 to +14 positions (Fig. 2). Such a rapid and highly repetitive reaction process occurring for a prolonged span of time is possible only if the sigma subunit remains tethered to the polymerase and can reform an open complex to initiate again and again. Taken together, these late abortive steps can account for the inordinately long escape half-lives measured for the various *N25anti(-A)* promoters (Table 3).

#### Step 3b: Template supercoiling provides a large driving force for completing the escape transition

While promoter release is necessary to begin the escape maneuvers, it is not sufficient to complete the escape process. For that, the upstream bubble collapse must occur with sufficiently favorable energetics to stabilize the downstream 11-bp elongation bubble (23, 74). Our kinetic measurements indicate that upstream bubble collapse is driven spontaneously by the upstream duplex DNA, which harbors a rewinding potential that is proportional to its length, but, more importantly, to the supercoiling status of the upstream DNA. Template supercoiling invariably stimulated promoter escape by facilitating upstream bubble rewinding in all four *N25-ITS* promoter variants. The circular plasmid DNA, being underwound as it is negatively SC, harbors a great deal of torsional stress energy that can be utilized to drive upstream bubble collapse (71, 78, 79) and complete the promoter escape transition.

## Summary/conclusion

Using single-cycle time-course transcription experiments performed under RNAP-limiting conditions, we determined the values of two parameters that govern productive RNA synthesis—(1), the productive fraction of open complexes formed at a promoter, and (2) the rate of escape (of the productive complexes)—on four *N25-ITS* promoters, each in three template conformations. We found that both parameters are subject to substantial modulation by the ITS and template supercoiling.

The ITS, as duplexed DNA, participates in the open complex formation process and can modulate the productive fraction in an as-yet-undefined sequence-dependent manner. As single strands, we found that the ITS controls the rate of productive transcription through both the abortive initiation phase and the promoter escape steps. Here, the sequence characteristics of the ITS relative to the open complex bubble strands determine the extent of this control. Thus, when comparing *N25(-C) versus N25anti(-A)*, the large differences in their escape kinetics and abortive transcription properties can be ascribed to the diametrically opposite sequence characteristics of their ITSs and whether each ITS agrees or deviates from the open complex bubble strands.

Given the functional involvement of the ITS in all facets of the transcription initiation process—demonstrated in the present study—plus the long-known fact that the ITS region is an integral part of the DNase I open complex footprint, it is appropriate for us to propose again that the ITS element (with associated sequence characteristics) be considered an integral unit in a promoter architecture (16). Its inclusion and consideration in promoter engineering design will facilitate the attainment of goals in many synthetic biology pursuits (80, 81).

In this study, the analysis of *N25anti(-A)* transcription kinetics in particular has revealed a rate-limiting step in a promoter escape mechanism and demonstrated the requirement of a driving force in each stage of initial transcription. During the abortive initiation stage, scrunching-induced stress energy harbored on the T-strand provides the driving force for promoter release. After promoter release, negative template supercoiling provides a large driving force for upstream bubble collapse and the formation of a stable 11-bp elongation bubble.

Our step-by-step description of a productive transcription mechanism not only showcases the multifold involvement of the ITS in the initiation phase up to the initiation–elongation transition but also provides an extension to the existing model of promoter escape. That is, promoter release creates an expanded bubble intermediate about to escape. The relative energetics of rewinding the upstream *versus* downstream portion of the expanded bubble determines the position of escape—again, an occurrence dependent on the sequence characteristics of the ITS.

A highlight of our study pinpoints the multiple ways negative template supercoiling can activate prokaryotic gene expression *in vivo*. Negative supercoiling of the template enhances the productive fraction of open complexes at a promoter and facilitates upstream bubble collapse for reaching the initiation–elongation transition. It most likely also facilitates the step-by-step upstream rewinding necessary during the elongation phase (74). The overall outcome is a higher level of productive complexes, making a much speedier escape into productive transcription.

Our investigation of *N25(-C) versus N25anti(-A)* shows how the two quantitative parameters of productive transcription can be modulated by the ITS *in vivo*. In *N25(-C)*, the native ITS evolved to facilitate the rate of escape while supporting a middling level of productive open complex formation. In *N25anti(-A)*, the *anti*-ITS yielded the highest productive fraction that then escapes at a greatly compromised rate. While the two parameters cannot be maximized simultaneously, the WT T5 *N25* promoter has opted for an enhanced rate of escape as a means to achieve optimal gene expression.

Finally, our approach can be applied to solve the long-standing paradox of T5 *N25* promoter activity *in vitro* and *in vivo* as compared with T7 *A1* and λ *P*_*L*_ (11). To resolve this paradox will require the determination of the quantitative parameters of productive transcription as studied in this work for all three promoters in their SC conformation.

## Experimental procedures

### Plasmid templates

The SC form of the single-cycle transcription templates was generated by cloning a 228-bp fragment (spanning −174 to +54) of *N25(-C)* or *N25anti(-A)* into the SalI/PstI sites of pSA508 vector (21); the resultant plasmids are 3559-bp in length and designated as pSAN1 or pSAN5, respectively. For *N25/A1(-U)* and *N25/A1anti(-G)*, a 233-bp fragment (spanning −174 to +59) was used; the resultant plasmids are 3564-bp in length and designated as pSAN6 and pSAN7, respectively.

### Transcription reactions

Time-course reactions were performed as follows. First, open complexes were formed with 80 nM template DNA and 40 nM RNAP, with or without a 10-fold molar excess of GreB, in 1X transcription buffer (40 mM Tris–HCl, pH 8.0, 10 mM MgCl_2_, 10 mM β-mercaptoethanol, 40 μg/ml acetylated bovine serum albumin), 200 mM KCl, for 10′ at 37 °C. At *t* = 0, an equal volume of a 2X nucleotide mixture made up in 1X transcription buffer and 200 mM KCl was brought to 37 °C and added to the open complex mixture. Time-point aliquots of 5 μl were sampled, mixed immediately with 5 μl of formamide loading dye (80% deionized formamide in 1X TBE, 10 mM Na_2_EDTA, 0.04% XC, and 0.04% Amaranth), and 4 μl of each sample was analyzed by high-percentage denaturing (23% (10:1)/7 M urea) PAGE. Final concentrations of the nucleotide mixtures were for *N25(-C)*: 100 μM ATP/GTP/3′-dCTP, 20 μM UTP with [α-^32^P]-UTP label; for *N25anti(-A)*: 100 μM GTP/CTP/3′-dATP, 20 μM UTP with [α-^32^P]-UTP label; for *N25/A1(-U)*: 100 μM ATP/GTP/3′-dUTP, 20 μM CTP with [α-^32^P]-CTP label; and for *N25/A1anti(-G)*: 100 μM ATP/CTP/3′-dGTP, 20 μM UTP with [α-^32^P]-UTP label. The FL-halted RNA on each template was C_30_, A_41_, U_33_, and G_35_, respectively.

For steady-state transcription reactions, we used 30 nM promoter DNA and 50 nM RNAP (60% active molecules) for all four templates. However, the nucleotide concentrations were different. For *N25(-C)* and *N25anti(-A)*, 100 μM A/C/GTP and 20 μM UTP with [α-^32^P]-UTP label at ∼10 cpm/fmol. For *N25/A1(-U)* and *N25/A1anti(-G)*, 100 μM of all four NTPs with [γ-^32^P]-ATP label at ∼10 cpm/fmol. Reactions were commenced by adding RNAP and incubated for 10 min at 37 °C.

### Analysis of transcription kinetics

For each RNA species in a time-course gel scan, we can obtain the IQV units associated with that RNA, plot directly in Kaleidagraph to determine its *t*_1/2_ (in minutes) and plateau level of synthesis (in IQV units). For FL RNA, *t*_1/2_ is related to *k*_*E*_ of the promoter being analyzed. The plateau level of FL RNA, however, needed further processing (see below) to obtain the (femto)molar amounts of productive complexes formed during each reaction.

### RNA quantitation and molar conversion

The molar conversion procedure was briefly described previously (82). For each time-course assay, we set up a radioactive nucleotide mixture of a certain specific radioactivity (cpm/fmol), make serial dilutions for scintillation counting to obtain an average cpm/μl value. We then generated two copies of a standard strip of Whatman 3 MM paper rectangles (3 mm × 10 mm) spotted with a fixed volume (10 μl) of the dilution series. One copy is taped to the gel for simultaneous exposure to the phosphor screen. The second copy is subjected to scintillation counting on the same day. After scanning, the standard strip yields a standard curve whose slope is the IQV/cpm conversion factor for that gel. Applying this conversion factor and the specific radioactivity, the (plateau) IQV value obtained for any RNA can be converted into (femto)molar values; this value is further multiplied by a factor of 2.5 (because only 40% of each time point sample was analyzed on PAGE) and divided by *N*, the number of [α-^32^P]-labeling nucleotide in each RNA species, to yield the total molar amounts of that RNA in each time point aliquot. For FL RNA, this number is indicative of the productive complexes formed during a time-course reaction. In our reaction set up, each time point aliquot of 5 μl contains 60 fmol of active RNAP molecules (83).

## Data availability

All data are contained within the article.

## Conflict of interest

The authors declare that they have no conflicts of interest with the contents of this article.
